# The marriage of coherent Raman scattering imaging and advanced computational tools

**DOI:** 10.1038/s41377-023-01160-z

**Published:** 2023-05-09

**Authors:** Walker Peterson, Kotaro Hiramatsu, Keisuke Goda

**Affiliations:** 1 Department of Chemistry, The University of Tokyo, Tokyo, 113-0033 Japan; 2 Research Center for Spectrochemistry, The University of Tokyo, Tokyo, 113-0033 Japan; 3 Department of Bioengineering, University of California, Los Angeles, CA 90095 USA; 4 Institute of Technological Sciences, Wuhan University, Wuhan, Hubei 430072 China; 5 LucasLand, Inc., Tokyo, 101-0052 Japan

**Keywords:** Microscopy, Raman spectroscopy

## Abstract

Coherent Raman scattering microscopy can provide high-contrast tissue and single-cell images based on the inherent molecular vibrations of the sample. However, conventional techniques face a three-way trade-off between Raman spectral bandwidth, imaging speed, and image fidelity. Although currently challenging to address via optical design, this trade-off can be overcome via emerging computational tools such as compressive sensing and machine learning.

Coherent Raman scattering (CRS) spectroscopy can provide label-free, chemically-specific molecular vibrational information of targets, making it valuable for chemical analysis and discrimination^1–13^. Extended to an imaging framework, Raman content can be used for molecular contrast across spatial dimensions, a potential boon to research in fields such as cell biology, where samples of interest are chemospatially diverse^7,14–30^. Although CRS-based imaging methods provide stronger signals than their conventional spontaneous Raman counterparts, reported CRS methods are far from satisfactory for certain applications, especially for tracking the complicated dynamics of live cells and tissues. Photon generation via CRS processes is fixed by nature, and basic spectroscopy research tends to tweak photon statistics along different axes of a given problem space. A consequence for CRS imaging is a three-way trade-off (Fig. 1) between the detected Raman spectral bandwidth, the imaging frame rate, and the image fidelity.

**Fig. 1:**
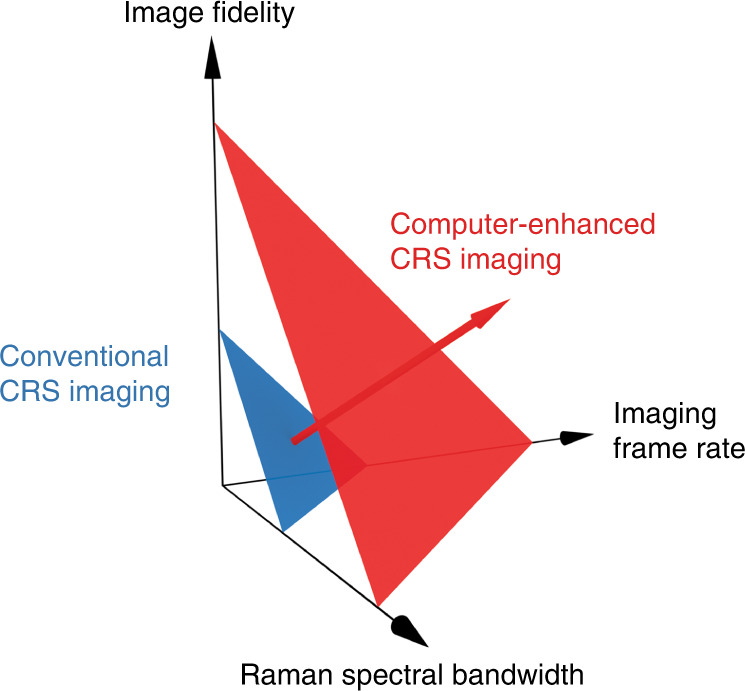
Three-way trade-off in CRS imaging. Conventional CRS imaging (blue) is now physically limited in terms of a trade-off between Raman spectral bandwidth, imaging frame rate, and image fidelity. This trade-off can be broken with computer-enhanced CRS imaging (red), where advanced computational tools extend the limits of optical design

For example, hyperspectral stimulated Raman scattering (SRS) spectra were obtained in as short as 5 µs^31^, but only over a narrow Raman spectral bandwidth of 200 cm^-1^. SRS imaging was demonstrated at a high imaging frame rate of 2 kHz, but images were limited to only four vibrational frequencies^16^. On the other hand, hyperspectral coherent anti-Stokes Raman scattering (CARS) imaging was shown over a much broader Raman spectral bandwidth of 3000 cm^-1^, but with a long pixel dwell time of 3.5 ms^19^. Likewise, Fourier-transform coherent anti-Stokes Raman scattering (FT-CARS) imaging was reported over a broad Raman spectral bandwidth of 800 cm^-1^ with a short pixel dwell time of 42 µs^22^. Although CARS imaging and FT-CARS imaging offer broader Raman spectral bandwidths than SRS imaging, it comes at the expense of speed. In a similar vein, in CRS imaging, higher-fidelity images generally require more pixels and take longer to obtain, which reduces the imaging frame rate. While careful optical design can be leveraged to maximize a technique’s performance by a particular metric, this tends to come at the expense of performance in one or both of the other two areas; the three-way trade-off in CRS imaging represents a physical limitation that is very difficult to overcome via optical design alone.

In a review article^32^ by Lin et al., the authors present a broad, well-informed summary of the emerging field of computational CRS imaging, where advanced computational techniques are applied to break through the three-way trade-off of conventional CRS imaging (Fig. 1). After an in-depth introduction of several techniques for CRS imaging and their physical limitations, Lin et al. provide clear explanations of how the various hurdles of the three-way trade-off can be addressed by appropriate computational methods. Specifically, the authors describe possible computation-based solutions to challenges of CARS spectral unmixing, CARS non-resonant background removal, sampling requirements in both time (FT-CARS) and frequency (SRS, CARS) domains, volumetric imaging, denoising, and more. The solutions include compressive sensing, principal component analysis, deep learning, spectral phasor, projection tomography, and many others.

Beyond its description of the current state of the art, Lin et al.’s review paper provides a forward-looking vision for the marriage of advanced computational techniques to CRS imaging. While the authors emphasize that care must be taken to apply computational methods appropriately, they also predict the increasing importance of computational solutions to the physical limitations of CRS imaging; computer-based advancement will be a key area of research, moving in tandem with development in instrument technology and core optics design. Moreover, we expect that this trend will accelerate the deployment of practical CRS imaging beyond the lab bench, into spheres where reliable, user-friendly solutions are much needed. For example, despite its potential value as a diagnostic tool, in vivo and ex vivo investigation of tissue via high-fidelity, rapid, broadband CRS imaging is still a challenge in real-world clinical settings, in part due to hurdles that can be addressed by advanced computational tools. In forensic science, computer-enhanced CRS imaging could relieve stress in judicial systems with limited resources and, importantly, ensure verity. Large-scale accessibility of systems for high-throughput, high-fidelity, broadband Raman imaging flow cytometry, cell sorting, and particle analysis could lead to a tremendous expansion of understanding in fields such as biology and environmental science, where large-scale label-free measurements at single-cell or -particle resolution are needed. In these and other practical settings, we expect a coming paradigm shift where advanced computational tools shoulder much of the burden of not only acquiring, managing, and processing, but also interpreting the huge data generated by CRS imaging with broad Raman spectral bandwidth, high image fidelity, and high imaging frame rate.
